# Computational analysis of LDDMM for brain mapping

**DOI:** 10.3389/fnins.2013.00151

**Published:** 2013-08-27

**Authors:** Can Ceritoglu, Xiaoying Tang, Margaret Chow, Darian Hadjiabadi, Damish Shah, Timothy Brown, Muhammad H. Burhanullah, Huong Trinh, John T. Hsu, Katarina A. Ament, Deana Crocetti, Susumu Mori, Stewart H. Mostofsky, Steven Yantis, Michael I. Miller, J. Tilak Ratnanather

**Affiliations:** ^1^Center for Imaging Science, The Johns Hopkins UniversityBaltimore, MD, USA; ^2^The Russell H. Morgan Department of Radiology and Radiological Science, The Johns Hopkins University School of MedicineBaltimore, MD, USA; ^3^Laboratory for Neurocognitive and Imaging Research, Kennedy Krieger InstituteBaltimore, MD, USA; ^4^Department of Neurology, The Johns Hopkins University School of MedicineBaltimore, MD, USA; ^5^Department of Psychiatry, The Johns Hopkins University School of MedicineBaltimore, MD, USA; ^6^Department of Psychological and Brain Sciences, The Johns Hopkins UniversityBaltimore, MD, USA; ^7^Institute for Computational Medicine, The Johns Hopkins UniversityBaltimore, MD, USA; ^8^Department of Biomedical Engineering, The Johns Hopkins UniversityBaltimore, MD, USA

**Keywords:** subcortical segmentation, computational anatomy, brain mapping, LDDMM

## Abstract

One goal of computational anatomy (CA) is to develop tools to accurately segment brain structures in healthy and diseased subjects. In this paper, we examine the performance and complexity of such segmentation in the framework of the large deformation diffeomorphic metric mapping (LDDMM) registration method with reference to atlases and parameters. First we report the application of a multi-atlas segmentation approach to define basal ganglia structures in healthy and diseased kids' brains. The segmentation accuracy of the multi-atlas approach is compared with the single atlas LDDMM implementation and two state-of-the-art segmentation algorithms—Freesurfer and FSL—by computing the overlap errors between automatic and manual segmentations of the six basal ganglia nuclei in healthy subjects as well as subjects with diseases including ADHD and Autism. The high accuracy of multi-atlas segmentation is obtained at the cost of increasing the computational complexity because of the calculations necessary between the atlases and a subject. Second, we examine the effect of parameters on total LDDMM computation time and segmentation accuracy for basal ganglia structures. Single atlas LDDMM method is used to automatically segment the structures in a population of 16 subjects using different sets of parameters. The results show that a cascade approach and using fewer time steps can reduce computational complexity as much as five times while maintaining reliable segmentations.

## Introduction

Computational anatomy (CA) methods have been at the forefront of neuroimaging studies of neurodevelopment and neurodegeneration. Software such as FSL (Jenkinson et al., 2012), AFNI (Cox, 2012), BrainVoyager (Goebel, 2012), Caret (Van Essen, 2012), FIASCO (Aguirre, 2012), SUMA (Saad and Reynolds, 2012), FreeSurfer (Fischl, 2012), and SPM (Ashburner, 2012) have been used to analyze structural and functional properties of the human brain via magnetic resonance images (MRI), diffusion tensor images (DTI), and functional MRI (fMRI) at a resolution of 1 mm^3^. In addition, pipelines for registering and visualizing hundreds of images have emerged. These include DTIStudio (Jiang et al., 2006), LONI (Dinov et al., 2006), ANTs (Avants et al., 2008), and 3D Slicer (Pieper et al., 2004). At the heart of these methods is the modeling of anatomical structures and their huge variation, an active field originally formalized as CA (Grenander and Miller, 1998). The main difficulty is the complexity of anatomical structures and the large variation between individuals. Here anatomical structures are represented as a collection of coordinate systems: landmark points, curves, surfaces, and sub-volumes. These structures are represented as deformable templates, with the space of anatomical images being the set generated by the group of diffeomorphic transformations acting on the template with associated probability laws, which describe their variation. The transformations are detailed so that a large family of shapes may be generated with the precise topology of the template maintained. In particular, our methods have demonstrated localized shape differences in multiple sub-cortical structures in neuroimaging studies of Alzheimer's Disease (Qiu et al., 2008a, 2009c), ADHD (Qiu et al., 2009b), Autism (Qiu et al., 2010), schizophrenia (Wang et al., 2008), and Tourette Syndrome (Wang et al., 2007).

The theoretical framework we have adopted is based on the large deformation diffeomorphic metric mapping (LDDMM) algorithm (Beg et al., 2005) and advances have been developed by others as well (Risser et al., 2010, 2011; Auzias et al., 2011). It also has been demonstrated that the best registration methods have high dimensional and diffeomorphic properties that have in common many of the properties incorporated into LDDMM (Klein et al., 2009, 2010). The LDDMM approach allows shape to be uniquely encoded by the vectors normal to the outline of the template (Miller et al., 2006). This property provides the basis of our shape analysis projects and is thus crucial in locating changes in multiple structures caused by disease leading to a better understanding of the effect of disease on neighboring structures.

To date, many MRI studies of subcortical gray matter nuclei have defined a single measure of structural volume. While this has the advantage of being quantitative, it does not give specific information about subregions of atrophying nuclei. This information would be useful in order to determine whether MRI morphometric results correlate with neuropathologic studies, to define better the subregional distribution of atrophy, and for correlation of pathologic changes with clinical features of the disease. Methods of statistical shape analysis have proved useful for studying normal age related changes in subcortical nuclei and for studying a number of other diseases (Bansal et al., 2007; Brun et al., 2009; Chung et al., 2010; Stein et al., 2011; van den Bogaard et al., 2011). Such analysis based on our LDDMM framework (Miller and Qiu, 2009) has proved useful for studying changes in subcortical nuclei in normal aging and several diseases (Csernansky et al., 1998, 2000, 2002; Wang et al., 2007; Qiu and Miller, 2008; Qiu et al., 2008b, 2009a,b,c,d, 2010). For example, analysis with the LDDMM based surface based morphometry (SBM) pipeline revealed heterogeneity of atrophy in subcortical structures in studies of Huntington's disease (Younes et al., 2012) and dementia (Miller et al., 2012).

So as the usage of neuroimaging software expands, it becomes critically important to provide novel analyses that go beyond volumes and/or other scalar quantities at the voxel or regional level. Specifically, the ability to segment subcortical structures in hundreds of scans which can then be used for shape analysis. However, segmentation accuracy and performance is limited by two complementary factors.

The first is the restriction to a single atlas as in MRIStudio which has become widely used in neuroimaging studies of MRI and DTI data as evidenced by meta-data search in Google Scholar. Currently, MRIStudio consists of three programs: DTIStudio (Jiang et al., 2006), DiffeoMap and ROIEditor. In particular, DiffeoMap is a program for image transformation based on LDDMM and ROIEditor uses the results of DiffeoMap to perform image analysis with respect to a single atlas both at the voxel and regional level. The restriction can be compounded by the wide anatomical variability in structures. For example, ventricles differ in shape and geometry in neuroimaging studies of aging and dementia (Reig et al., 2007). Further, proximity of sub-cortical structures can affect the accuracy and performance of automated segmentation algorithms resulting in conflicting results at the volumetric level (Heckemann et al., 2010). Here we explore the incorporation of multi-atlases to drive the segmentation of subcortical structures which should lead to improved statistical significance in shape analysis even from a small sample.

The segmentation problem, which relies on learning anatomical information from pre-segmented training datasets, is usually handled in the Bayesian framework by solving a maximum a posteriori (MAP) estimation problem. The information of multiple pre-segmented training datasets can be combined naturally in the Bayesian framework. Compared with single atlas based segmentation, multi-atlas based segmentation has been shown to be more powerful and more accurate (Rohlfing et al., 2004; Heckemann et al., 2010; Langerak et al., 2010; Lotjonen et al., 2010). More recently we have demonstrated the use of LDDMM with multiple atlases in which both the diffeomorphic coordinate change as well as the atlas being selected is unknown. Such an approach has been shown to be efficient in mediating large deformations in the context of building reliable segmentations of both subcortical and cortical structures (Tang et al., 2012).

This leads to the second problem which is the computational complexity of LDDMM which can be compounded by multiple atlases. By default, LDDMM computes the geodesic along the Riemannian manifold from the template to the target in ten uniform time steps (Beg et al., 2005; Ceritoglu et al., 2009; Tward et al., 2011). As such it takes about 30 min to map one whole brain at 1 mm^3^ resolution using a parallelized implementation on Intel Xeon CPU E5530 at 2.40GHz with 16 cores. By evolving the flow field over time, it allows for large deformations which come at a cost in terms of computational resources. In the multiple atlas setting, the computational requirements increase as order number of atlases. In an attempt to overcome the computational complexity, LDDMM has inspired alternative diffeomorphic implementations image registration algorithms such as DARTEL (Ashburner, 2007), Diffeo DEMONS (Vercauteren et al., 2009) and Spherical Demons (Yeo et al., 2010) to name but a few. These methods essentially compute the mapping in one time step to generate a “stationary velocity field” in the spirit of exponential maps for finite dimensional groups. While these mappings no longer satisfy the conservation law and therefore metric property giving rise to complete encoding of the diffeomorphic flow of the shape in the initial tangent vector (Miller et al., 2006), their diffeomorphic features make them powerful alternatives for generating segmentations which are accurate. Then metric structure can be derived from the segmentations as demonstrated (Miller and Qiu, 2009). By reformulating the optimization problem, LDDMM can be recast as EPDiff, i.e., an initial value problem (IVP) in which geodesic shooting is used to evolve the initial momenta to match with the target shape (Miller et al., 2006; Marsland and McLachlan, 2007; Ashburner and Friston, 2011; Vialard et al., 2012).

This paper examines performance and complexity of LDDMM with reference to atlases and parameters. A natural way is to examine tradeoff of computational complexity vs. segmentation reliability. Hence we examine segmentations of multiple sub-cortical structures from ongoing large scale neuroimaging studies which are different and more complex than those previously studied (Tang et al., 2012, 2013).

## Methods and data

### Data

The first dataset included thirty whole brain, high resolution T1-weighted 3D-volume MPRAGE images (matrix size = 256 × 256, echo time = 3.76 ms, repetition time = 7.99 ms, field of view = 256 mm, slice thickness = 1.0 mm) acquired from a 3T Philips Gyroscan NT scanner (Royal Philips Electronics, Amsterdam, The Netherlands). The dataset included 13 healthy subjects (mean age 10.42 years old; 5 males, and 8 females), 6 male subjects with autism spectrum disorder (ASD) (mean age 9.74 years old) and 11 subjects diagnosed with Attention Deficit/Hyperactivity Disorder (ADHD) (mean age 10.2 years old; 4 males, and 7 females). The second dataset included sixteen whole brain, high resolution T1-weighted 3D-volume MPRAGE images. Eight were healthy subjects (mean age 20.75 years old; all female) acquired from a Philips Intera 3T scanner (Royal Philips Electronics, Amsterdam, The Netherlands; matrix size = 256 × 256, echo time = 3.8 ms, repetition time = 8.1 ms, field of view = 256 mm, slice thickness = 1.0 mm). Four were diagnosed with primary progressive aphasia (mean age 68 years old) acquired from a 1.5T Philips Gyroscan NT (Royal Philips Electronics, Amsterdam, The Netherlands; matrix size = 256 × 256, echo time = 32 ms, repetition time = 6.85 ms, field of view = 230 mm, slice thickness = 1.0 mm). The other 4 were subjects with Alzheimer's disease (AD) (mean 75.6 years old) acquired from a 1.5T Philips Gyroscan NT (Royal Philips Electronics, Amsterdam, The Netherlands; matrix size = 256 × 256, echo time = 3.2 ms, repetition time = 6.9 ms, field of view = 240 mm, slice thickness = 1.2 mm). All data were resampled to isotropic 1 mm^3^ voxel resolution and image size of 181 × 217 × 181.

### LDDMM

Given an atlas image *I*_0_ and a target image *I*_1_, which can be represented as functions *I*_0_, *I*_1_:Ω ⊆ *R*^3^ → *R* on the spatial domain Ω ⊆ *R*^3^, the LDDMM algorithm (Beg et al., 2005; Ceritoglu et al., 2009) computes a diffeomorphic transformation φ:Ω → Ω between these images such that *I*_1_ = *I*_0_·φ^−1^. The diffeomorphism φ = ϕ_1_ is defined as the end point of the curve φ = ϕ_*t*_, t ∈ [0, 1] satisfying the ordinary differential equation ${\dot{\phi }}_{t}=v_{t}\left(\phi_{t}\right)$, where ϕ_0_ = *Id* is the identity transformation and *v*_*t*_:Ω → *R*^3^, *t* ∈ [0, 1] is the time dependant velocity vector field of the flow of deformation.

The diffeomorphism φ is calculated as: φ = ϕ_1_ = ∫^1^_0_*v*_*t*_ (ϕ_*t*_)*dt* with ϕ_0_ = *Id*, where the optimal *v*_*t*_ is estimated by solving the variational problem:

(1) $$\hat{v}=\underset{v:{\dot{\phi }}_{t}\;=\;v_{t}\left(\phi_{t}\right)}{\arg\min}\left(\int_{0}^{1}{\left\|Lv_{t}\right\|}_{L^{2}}^{2}dt+\frac{1}{\sigma^{2}}{\left\|I_{0}\;\cdot \;\phi_{1}^{-1}-I_{1}\right\|}_{L^{2}}^{2}\right)$$

Theoretically, the minimizer of equation (1) results in a geodesic path for the curve ϕ_*t*_ in the space of diffeomorphisms. To ensure that the solution lies in the space of diffeomorphisms, smoothness is achieved by defining the operator *L* as: *L* = −α∇^2^ + γ*I*_3 × 3_, where ∇^2^ is the Laplacian operator.

In LDDMM, steepest gradient descent approach is used to perform the minimization in equation (1) and the velocity field at each gradient descent iteration *k* is updated with

(2) $$v_{t}^{k+1}=v_{t}^{k}-\varepsilon (\nabla_{v_{t}^{k}}E_{t}),$$

where ∇_*v*_ *E*_*t*_ is the gradient of the cost in equation (1).

(3) $$\nabla_{v}E_{t}=2v_{t}-K\text{*}\left(\frac{2}{\sigma^{2}}\left|D\phi_{t,\;1}^{v}\right|\nabla J_{t}^{0}\left(J_{t}^{0}-J_{t}^{1}\right)\right),$$

where ϕ_*s*, *t*_ = ϕ_*t*_ · ϕ^−1^_*s*_, *J*^0^_*t*_ = *I*_0_ · ϕ_*t*, 0_, *J*^1^_*t*_ = *I*_1_ · ϕ_*t*, 1_, |*D*ϕ^v^_*t*, 1_| is the determinant of the Jacobian matrix, *K* = (*L*^†^*L*)^−1^ and ^*^ is the convolution operation.

In the numerical implementation of LDDMM, the time parameter *t* of the flow is discretized with a fixed total number of timesteps T, where *T* = 10 is selected as the default value in general. Selection of a smaller T causes gradient descent to terminate with a higher final mismatch error ‖*I*_0_ · φ^−1^ − *I*_1_‖^2^_*L*^2^_ between the registered atlas image and the target image. In this article *T* = 1 is chosen to approximate the small deformation setup for comparison with *T* = 10.

The background space of the images is represented with Ω = [0, 1]^3^ and the convolution operation in equation (3) is calculated in Fourier domain. The operator *K* acts as a low pass filter at each iteration of gradient descent and the parameters α and γ controls the amount of smoothing and the elasticity of the deformation. Selection of these parameters depends on the size of the deformation (in pixels) necessary to register the features of the atlas image to the features of the target image. Resampling the atlas and target images to same resolution and doing an initial rigid or affine alignment as a preprocessing step for LDDMM allows the selection of α/γ in the range of 0.01–0.001 to accurately register the features of MR brain data with a typical resolution in the range of 0.5–2 mm/voxel. As α/γ decreases in the 0.01–0.001 range, the image matching quality increases and the deformations become more local and elastic (Beg et al., 2005; Ceritoglu et al., 2009; Tward et al., 2011).

In the cascading implementation of LDDMM (Ceritoglu et al., 2009), The final diffeomorphic deformation φ between images *I*_0_and*I*_1_ is calculated by combining deformations φ_1_, φ_2_, …, φ_*n*_ and φ = φ^−1^_1_ · φ^−1^_2_ · … · φ^−1^_*n*_ where (i) φ_1_ is calculated between *I*_0_ and *I*_1_, (ii) φ_2_ is calculated between *I*_0_ · φ^−1^_1_ and *I*_1_, (iii) φ_3_ is calculated between *I*_0_ · φ^−1^_1_ · φ^−1^_2_ and *I*_1_. In each step, the regularization parameter γ is fixed to be 1 and α is decreased to ensure that final registration is more robust and does not converge to an apparently incorrect solution compared to computing φ directly between *I*_0_ and *I*_1_ using very small α value (Ceritoglu et al., 2009) directly without cascading. The cascading implementation does not calculate a geodesic trajectory between the images and the mathematical properties of LDDMM such as metric distance and the encoding of the diffeomorphic flow of the shape with the initial tangent vector are not retained.

It is worth noting that geodesic trajectories can be obtained via multi-scale approaches (Risser et al., 2011; Vialard et al., 2012) where a weighted sum of Gaussian kernels is used to define the operator *K* in equation (3) control the elasticity of the deformation at each scale.

### Segmentation via single-atlas

**Figure 1:**
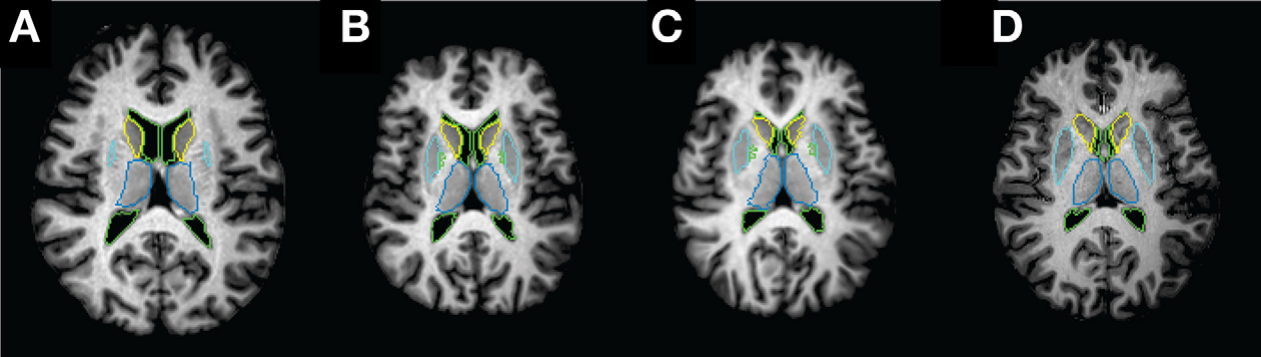
**Example axial slices of atlas and subject T1 weighted MR image and the boundaries of their ROI labels at each registration step. (A)** Original atlas image and atlas ROIs; **(B)** atlas image and ROIs after linear AIR transformation; **(C)** atlas image and ROIs after AIR and LDDMM transformations; **(D)** subject image and subject ROIs.

The single-subject atlas is the JHU-DTI-MNI atlas (Oishi et al., 2009), which is a single-subject template in the ICBM-DTI-81 space. The template has an isotropic 1 mm^3^ voxel resolution and image size of 181 × 217 × 181. Histogram matching was applied to the atlas and subject image, followed by initial affine alignment of the atlas onto the subject via AIR (Woods et al., 1998a,b). MRIStudio was then used to register the subject to the atlas. The resulting diffeomorphism was used to transfer the ROIs defined in the atlas to the subject space resulting in ROIs segmented in the subject space. This procedure is illustrated in Figure 1. All experiments were performed using Intel Xeon CPU E5530 at 2.40GHz with 16 cores.

### Segmentation via multi-atlas fusion

The algorithm for multi-atlas segmentation using LDDMM (Tang et al., 2012, 2013) is briefly summarized as follows. Assume there are multiple MRI atlases, each of which contains a collection of locally-defined charts obtained from manually segmented structures. A MAP approach is used to estimate the high dimensional segmentations from the class of generative models representing the observed MRI which is assumed to be a Gaussian random fields conditioned on the atlas charts and diffeomorphic change of coordinates of each chart. The charts and their diffeomorphic correspondences are unknown and viewed as latent or hidden variables. The expectation-maximization (EM) algorithm yields the likelihood-fusion equation which is maximized by the a posteriori estimator of the segmentation labels. The fused likelihoods are modeled as conditional Gaussian random fields with mean fields a function of each atlas chart under its diffeomorphic change of coordinates onto the target. The conditional-mean in the EM algorithm specifies the convex weights with which the chart-specific likelihoods are fused. The multiple atlases with the associated convex weights imply that the posterior distribution is a multi-modal representation of the measured MRI. As with the single-atlas segmentation, the resulting diffeomorphism was used to transfer the ROIs to the subject space resulting in ROIs segmented in the subject space.

### Comparison metrics

Automated segmentation of subcortical structures was compared with manual segmentations of the same subjects. The manual segmentations are based on anatomical definitions (Wang et al., 2007; Qiu et al., 2009b, 2010) with the assistance of an atlas (Mai et al., 1997); for details see http://caportal.cis.jhu.edu/protocols. Manual segmentation was performed with the open source software Seg3D which follows the radiological convention for displaying images.

Accuracy for each ROI was quantified by three metrics. The first is the kappa statistic (Cohen, 1960) is defined by κ = (*p*_agree_ − *p*_random_)/(1 − *p*_random_)where *p*_agree_ is the fraction of voxels in which the given segmentation agrees with the manual segmentation, and *p*_random_ is the fraction one would expect by random chance (based only on the volumes of foreground and background). Note that κ is biased by the volume size of the structure; generally, the bigger the structure, the higher the kappa statistic is. For brain structures, a value of κ = 0.8is considered quite good. The second is the volume error which quantifies volume difference between two label defined by *VD*(*L*_*A*_, *L*_*M*_) = 100|*V*(*L*_*A*_) − *V*(*L*_*M*_)|/*V*(*L*_*M*_) where *V*(*L*_*A*_)and *V*(*L*_*M*_) are respectively the volume size of the automated and manual segmentation. The third is the *L*_1_ misclassification error (Miller et al., 2000) defined by *L*_1_ = |*A* ∪ *M*| − |*A* ∩ *M*|/|*A* ∪ *M*|/2 where *A* and *M*respectively are the set of automated and manual labeled voxels.

### Statistics

In the first experiment, One-Way ANOVA was performed on κ and *L*_1_ from the automated and manual segmentations. Multiple comparisons were conducted using *Tukey's HSD* (honestly significant difference) test between pair wise groups. In the second experiment, two statistical analyses were performed to examine the effects of LDDMM parameters—T and α —on the segmentation accuracy of single-atlas based registration. In the first analysis, mean*L*_1_ and volume errors were calculated for each subjects by averaging the errors of the segmented ROIs for that subject. Then Two-Way ANOVA was conducted to examine the effects of changing T and α on mean *L*_1_ and volume error. Multiple comparison tests using *Tukey's HSD* was carried out after ANOVA. In the second analysis, the effect of T on the errors for each ROI was analyzed separately via one sided paired samples *t*-test between *T* = 1 and *T* = 10 results for given α.

## Results

**Figure 2:**
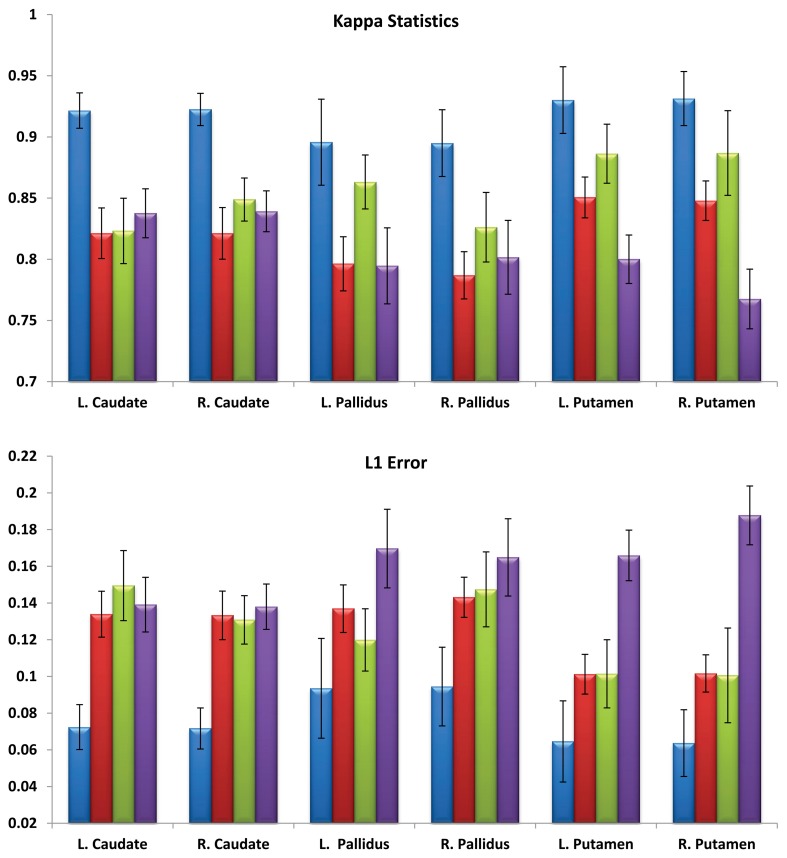
**Mean and standard deviation of mean Kappa statistics (top) and mean *L*_1_ errors (bottom) for left and right pairs of caudate, putamen and pallidus in 30 subjects via four different segmentation methods: multi-atlas (blue), single-atlas (red), FSL (green), and FreeSurfer (purple)**.

**Figure 3:**
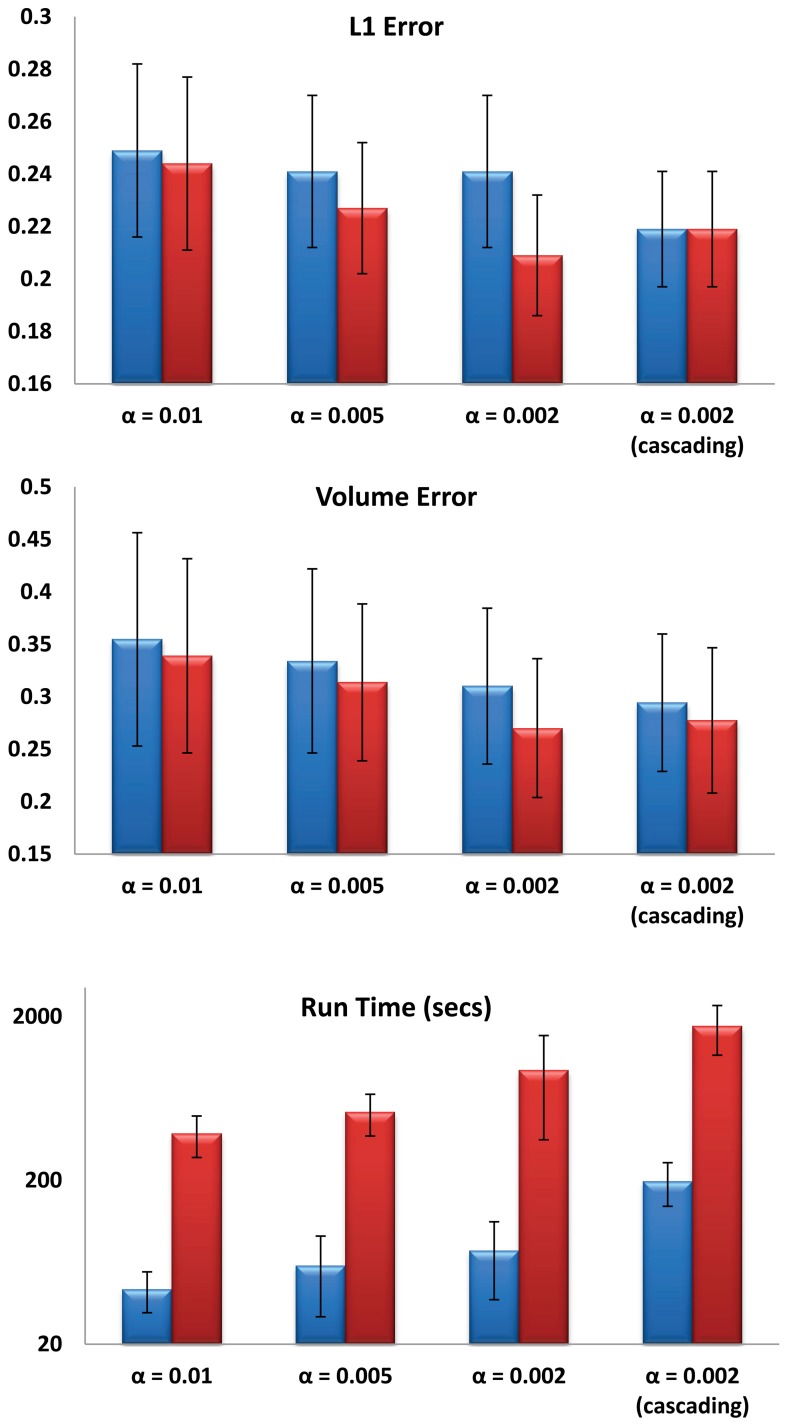
**Mean and standard deviation of mean *L*_1_ error (top), mean volume error (middle) and LDDMM running times (bottom) calculated from a population of 16 subjects for different values of α, *T* = 1 (blue) and *T* = 10 (red)**. Mean errors were calculated using 7 pairs of caudate, putamen, pallidus, amygdala, hippocampus, thalamus, and ventricle for each subject.

For the first experiment, multi-atlas segmentation with cascading values of α = 0.01, 0.005, 0.002 and *T* = 10 was compared with single-atlas segmentation with the same values of α and T, FSL (version 5.0) and FreeSurfer (version 5.2.0). Six structures were analyzed (left and right pairs of caudate, putamen and globus pallidus). Mean and standard deviation of kappa overlap and *L*_1_ errors are shown in Figure 2. One-Way ANOVA results (*F*_L.caudate_ = 155.57, *F*_R.caudate_ = 196.8, *F*_L.globuspallidus_ = 95.45, *F*_R.pallidus_ = 96.85, *F*_L.putamen_ = 182.73, *F*_R.putamen_ = 228.23, *df* = 3, *P* < 0.001) for kappa overlap and (*F*_L.caudate_ = 163.08, *F*_R.caudate_ = 187.79, *F*_L.globuspallidus_ = 73.89, *F*_R.globuspallidus_ = 75.90, *F*_L.putamen_ = 186.88, *F*_R.putamen_ = 244.95, *df* = 3, *P* < 0.001) and the multiple comparisons test with Tukey's HSD showed that for each of six structures considered, the automated segmentations obtained from multi-atlas LDDMM are statistically more accurate than those from the other three methods. For the second experiment, the following combinations were used: α = {0.01, 0.005, 0.002, 0.002with cascading} and *T* = {1, 10}. So, a total of (4 α-values) × (2 *T*-values) = 8 different automated segmentations were calculated for each subject using LDDMM single-atlas segmentation. Fourteen structures (left and right pairs of caudate, putamen, globus pallidus, hippocampus, amygdala, thalamus, ventricle) were analyzed. Figure 3 (top and middle rows) shows the mean and standard deviation of these errors computed over 16 subjects. There was no significant interaction between the effects of T and α, (*F* = 1.6785, *df* = 3, *P* = 0.17527). Simple main effects analysis showed that the effects of both α (*F* = 7.5801, *df* = 3, *P* = 0.00011) and T (*F* = 9.3323, *df* = 1, *P* = 0.0028) are statistically significant. A similar analysis on mean volume error showed that there was no significant interaction between the effects of T and α, (*F* = 0.16, *df* = 3, *P* = 0.9230). Simple main effects analysis of the mean volume error showed that the effect of α (*F* = 4.1938, *df* = 3, *P* = 0.0073) was statistically significant but the effect of T (*F* = 2.7054, *df* = 1, *P* = 0.1026) was not statistically significant. Multiple comparisons test with Tukey's HSD, showed that there is a significant difference (*P* < 0.01) between using a high α (0.01) and low α (0.002 with and without cascading). Tables 1, 2 shows the statistically significant differences (*P* < 0.01) for *L*_1_ error and volume error respectively for the results at *T* = 1 and *T* = 10 at a significance alpha level of 0.01.

**Table 1:** **For each ROI and for each α in LDDMM, the *L*_1_ error results for *T* = 1 and *T* = 10 were compared using one sided paired sample *t*-test**.

	**Paired *t*-test results for *L*_1_ error**			
	**α = **0.01****	**α = **0.005****	**α = **0.002****	**α = **0.002** (cascading)**
Left amygdala				
Left caudate		T1 > T10	T1 > T10	
Left hippocampus				
Left globus pallidus				
Left putamen			T1 > T10	
Left thalamus	T1 < T10		T1 > T10	T1 > T10
Left ventricle	T1 > T10	T1 > T10	T1 > T10	T1 > T10
Right amygdala				
Right caudate		T1 > T10	T1 > T10	
Right hippocampus				
Right globus pallidus			T1 > T10	
Right putamen			T1 > T10	
Right thalamus				
Right ventricle	T1 > T10	T1 > T10	T1 > T10	T1 > T10
Number of ROIs with significant differences	3	4	8	3

Statistically significant (p < 0.01) error differences between large deformation map (T = 10) and small deformation map (T = 1) are indicated by either “T1 > T10” or “T1 < T10.”

**Table 2:** **For each ROI and for each α in LDDMM, the volume error results for *T* = 1 and *T* = 10 were compared using one sided paired samples *t*-test**.

	**Paired *t*-test results for volume error**			
	**α = **0.01****	**α = **0.005****	**α = **0.002****	**α = **0.002** (cascading)**
Left amygdala				
Left caudate	T1 < T10	T1 < T10		
Left hippocampus		T1 < T10	T1 < T10	
Left globus pallidus				
Left putamen				
Left thalamus	T1 < T10	T1 < T10		T1 > T10
Left ventricle	T1 > T10	T1 > T10	T1 > T10	T1 > T10
Right amygdala				
Right caudate	T1 < T10	T1 < T10		
Right hippocampus		T1 < T10	T1 < T10	T1 < T10
Right globus pallidus		T1 < T10	T1 < T10	
Right putamen				
Right thalamus	T1 < T10			
Right ventricle	T1 > T10	T1 > T10	T1 > T10	T1 > T10
Number of ROIs with significant differences	6	8	5	4

Statistically significant (p < 0.01) error differences between large deformation map (T = 10) and small deformation map (T = 1) are indicated by either “T1 > T10” or “T1 < T10.”

Figure 3 (bottom row) shows the average running times of LDDMM calculations for any selection of α and T for the populaton of 16 subjects. Computational complexity of each gradient descent iteration in LDDMM depends on T linearly. Smaller α also increases the computation time.

## Discussion

In this paper, we examined the computational complexity in mapping multiple atlases for segmentation of subcortical structures—caudate, putamen, and globus pallidus. Our results show that a cascade of α parameters and selecting fewer time steps can reduce computational complexity as much as five times while obtaining reliable segmentations.

We also compared the segmentations with two other methods—FSL and FreeSurfer. LDDMM based segmentation is capable of achieving comparable or superior accuracy as measured by either the kappa statistic or the *L*_1_ error. Extending the single-atlas LDDMM based segmentation to multiple atlases, we found that the segmentation accuracy was significantly increased. The even split between male and female subjects in the dataset of 30 subjects suggests that our algorithm is robust with respect to gender. Then in terms of pathological variability, the datasets consisted of control subjects, subjects with autism, as well as subjects with ADHD. So our multi-atlas LDDMM segmentation algorithm can be applied to a wide variety of subjects. This is crucial for clinical neuroimaging studies since the ultimate goal of any segmentation algorithm is to be able to accurately delineate brain structures with different diseases. The performance of multi-atlas LDDMM, in terms of segmenting subcortical structures of a different dataset, has been compared with STAPLE which is a classic label-fusion based segmentation method (Warfield et al., 2004). The segmentations were demonstrated to be superior to those from STAPLE (Tang et al., 2012). The high segmentation accuracy obtained from multi-atlas LDDMM is at the cost of more running time, given that we need to first segment multiple atlases and we need to do multiple registrations. This issue led to the second experiment—analyzing the two parameters α and T that determines the speed, memory requirement and accuracy of LDDMM, trying to find the optimal α and T that will speed up the registration without affecting segmentation accuracy.

In LDDMM, α and T are the two most important parameters that affect the smoothness of the computed diffeomorphism and the registration accuracy between the images that are registered. The results of the second experiment showed that decreasing α, decreased the mean and standard deviation of the errors and the results are statistically significant. Increasing T and using a large deformation setup instead of a small deformation setup also decreased the errors (Table 1) but results are less significant. Therefore, in general a small α should be selected for better registration. When each ROI is considered separately (first 3 columns of Table 1 and Table 2), using a large deformation map (*T* = 10) instead of a small deformation map (*T* = 1) decreased the errors further with smaller α for given ROIs. Instead of using a single α to compute the final transformation with LDDMM, using a cascading scheme and decreasing α gradually to compute the final transformation allows the selection of a smaller T (last column of Table 2 and Table 2). This result is very important especially for multi-atlas LDDMM where a large number of LDDMM computations are necessary for superior segmentation accuracy. Thus in the extreme case of selecting *T* = 1 leads to the mappings that capture only small deformations while increasing T with finer discretization of time flow leads to the mappings that capture larger deformations and are more smooth.

Considered by some to be the “state of art” registration method (Ashburner and Friston, 2011), LDDMM has been shown to yield accurate segmentation of subcortical structures in diverse datasets. Accuracy is obtained at a cost of computational complexity. As in the original pipeline (Miller and Qiu, 2009) in which segmentations were used to drive geodesic representations from which momentum and statistics were performed, the cascade approach described here allows us to generate intermediate analyses such as segmentations in a computationally efficient way. This is extremely important in our multi-atlas formulation with complexity *O*(*N*), where *N* is the number of atlases. Thus via MRIStudio, LDDMM offers the potential for more precise and sensitive analysis of anatomical structures in neuroimaging studies.

### Conflict of interest statement

The authors declare that the research was conducted in the absence of any commercial or financial relationships that could be construed as a potential conflict of interest.
